# Hospitals Visit Intention and Visit Decision: How the Role of Viral and Word of Mouth Marketing?

**DOI:** 10.3389/fpubh.2022.948554

**Published:** 2022-07-13

**Authors:** E. Rahmat Taufik, Samsurijal Hasan, Titin Titin, Faurani Santi Singagerda, Ella Anastasya Sinambela

**Affiliations:** ^1^Department of Management and Business, Fak Ekonomi dan Bisnis Univ Sultan Ageng Tirtaya Banten, Serang, Indonesia; ^2^Department of Management, STIE Bangkinang, Pekanbaru, Indonesia; ^3^Department of Management, Universitas Islam Lamongan, Lamongan, Indonesia; ^4^Department of Management, Faculty of Economics and Business, Institute of Informatics and Business Darmajaya, Lampung, Indonesia; ^5^Department of Management, Universitas Sunan Giri Surabaya, Sidoarjo, Indonesia

**Keywords:** visit intention, visit decision, hospitals, word of mouth marketing, viral marketing

## Introduction

In the era of health disruption 4.0, there are still many hospitals and health care facilities that face various challenges. The main challenges are felt in terms of unclear laws and regulations and the lack of harmonization of regulations between related ministries. In addition, from internal factors, the lack of openness, motivation, and good knowledge management on the part of hospital management, medical service doctors, and IT teams in the organization also need to be addressed. Hospitals need to be motivated to immediately implement SIMRS in their management in order to realize optimal data integration on a national scale. The problem of using big data, data security and protection, data privacy, and the use of cloud computing systems is also one of the issues that is quite challenging to understand and apply in business.

According Kristijono et al. (1) the activity Marketing a business using the internet is called e-marketing. In e-marketing business marketing activities, one of the things that is currently developing is viral marketing or also called WOM (Word of Mouth Marketing). Viral marketing is the internet's version of the use of word of mouth marketing, which is closely related to a message or marketing method that is so contagious or chained that consumers want to pass it on to others. According to Manuel et al. (2) viral marketing as a marketing effort that utilizes the power of news whose marketing method is very contagious or chain. In the world of the internet, The spread is implemented through social media, such as Facebook, Instagram, YouTube, Twitter, Google. Viral marketing is a marketing technique that uses social networks to achieve a specific marketing goal. The key to viral marketing is getting website visitors and recommending them to those who will be considered interested. This social media has a display message feature on the wall where everyone who befriends or follows the sender can see what both known and unknown people have to say. Thus, whatever the sender sends will be seen by all those who are friends with him. Indirectly, the more followers there are, the more news or products uploaded to social media will become viral, with the virality of the product, it will affect the price and value of a product's prestige.

The healthcare industry is entering an era of digital innovation where patients are looking for services that can directly answer their needs because they are limited by their daily activities. According to DMN3 data, consumers looking for medical information on the internet, 47% seek information about doctors, 38% for hospitals and health facilities, and 77% to book a health check-up schedule. Based on these facts, it is necessary for the hospital management team to find out the needs of target consumers or patients and incorporate them into a digital system (e.g., ease of access using a smartphone). This market need is being exploited by several health technology companies, which are currently growing in the community.

According to Banuara (3) and Wijaya et al. (4) define if its use is offline then it is called WOM and if its use is online then that is called viral marketing or electronic word of mouth. Viral marketing is considered the most effective medium to attract people's attention to be moved and care for others because of its very wide reach and impact and at this time almost everyone is very skilled in operating the functions of their gadgets and on average at this time these almost all have social media accounts. In addition, this non-profit organization is not too many organizations. people are interested in and thus the purpose of the discussion to be achieved is to find out whether viral marketing is effective as a social marketing medium in increasing public awareness to play an active role or want to be involved in non-profit organizations that care about others or care about the environment.

## E-WOM

According to Wijayaa et al. (4) and Then and Felisa (5) eWOM or electronic word of mouth is an important aspect of marketing programs in developing consumer expression of brands. The effect of online branding shows that current buying is due to the strong role of eWOM in building and activating brand image. According to Renwarin (6) and Sitorus et al. (7) good word of mouth through the following eight dimensions: Platform, Concern for Other, Economic Intensive, Helping Company, Expressing Positive Emotions, Venting Negative Feelings, Social Benefits, and Advice Seeking.

### Viral Marketing

According to Sitorus et al. (7) viral marketing is a marketing technique that utilizes social networks, both through cyberspace and offline which aims to be able to convey messages and advertisements to consumers in the market. If it can be defined simply, then virtual marketing can be said as a form of marketing process that can be done online or offline regarding the products/services you have to consumers. According to Wijayaa et al. (4) and Purwanto et al. (8) this marketing method will be carried out by the provider of goods/services which will be conveyed through information whether it's a website, video, newspaper, ebook, and others. Viral marketing is also part of the portfolio marketing strategy section. If simplified again, viral marketing in offline form can be in the form of delivering information by word of mouth. Meanwhile, if you go online, you can use social media which is currently a trend in society.

### Visit Interest

Interest is the driving force that causes a person to pay attention to other people or other objects. Interest is one source of motivation for a person to do activities that are liked According to Ngelyaratan et al. (9) which will have an impact on increasing market share. There are 3 (three) factors that can generate a person's interest, namely internal encouragement factors, social motive factors and emotional factors. Basically, visiting interest is the feeling of wanting to visit an interesting place or area to visit. In this case, the theory of interest in visiting is taken from the theory of buying interest in a product, so that in several categories, interest in visiting can be applied from the model of buying interest. The following is an explanation of the theory of visiting interest. Interest is the drive to motivate someone to take action. According to Schiffman and according to Ngelyaratan et al. (9) argues that buying interest is a psychological activity that arises because of feelings and thoughts about a desired product or service. Based on some of these opinions, it can be concluded that interest in visiting is a statement of someone's desire to buy a product or service.

### Visit Decision

According to Renwarin (6) and Sitorus et al. (7) consumer buying behavior is the buying behavior of individuals, namely final consumers who buy goods and services for personal consumption. So consumer behavior here means about the thoughts, considerations, actions and feelings of consumers when choosing a product to satisfy their needs and desires. According to Hubner et al. (10) and Juwaini et al. (11) the decision is a selection of two or more of the available alternatives. In this case, alternative options must be available when the decision-making process is carried out. Alternative choices are used as comparisons or references when a decision will be made. According to Wijayaa et al. (4) and Then and Felisa (5) the stages in purchasing decisions made by consumers are Information Search, then consumers are moved to seek more information related to their needs.

## Result: Discussion and Opinion

### E-WOM Relationship and Visiting Interest

E-WOM has a positive and significant effect on visiting interest. An increase in the E-WOM variable will encourage a significant increase in the visiting interest variable and a decrease in the E-WOM variable will encourage a significant increase in the visiting interest variable. The results of this study are supported by research conducted by Wijaya et al. (4), Then and Felisa (5), Renwarin (6), Sitorus et al. (7), and Purwanto et al. (12).

### Viral Marketing Relationship and Interest in Visiting

Viral marketing has a positive and significant effect on Visiting Interest. An increase in the Viral marketing variable will encourage a significant increase in the Visiting Interest variable and a decrease in the Viral marketing variable will encourage a significant increase in the Visiting Interest variable. This study has similar results with research conducted by Banuara (3), David et al. (13), and Haudi et al. (14).

### E-WOM Relationship and Visiting Decisions

E-WOM has a positive and significant effect on visiting decisions. An increase in the E-WOM variable will encourage a significant increase in the visiting decision variable and a decrease in the E-WOM variable will encourage a significant increase in the visiting decision variable. The results of this study are supported by the research of Wijaya et al. (4) and Then and Felisa (5).

### Viral Marketing Relationships and Visiting Decisions

Viral marketing has a positive and significant effect on visiting decisions. An increase in the Viral marketing variable will encourage a significant increase in the visiting decision variable and a decrease in the Viral marketing variable will encourage a significant increase in the visiting decision variable. The results of this study are supported by research conducted by Wijaya et al. (4), Then and Felisa (5), Renwarin (6), Sitorus et al. (7), and Purwanto et al. (12).

### Interest in Visiting and Visiting Decisions

Visit interestt has a positive and significant effect on visiting decisions. An increase in the variable interest in visiting will encourage a significant increase in the visiting decision variable and a decrease in the variable interest in visiting will encourage a significant increase in the variable in the decision to visit. The results of this study are supported by research conducted by Wijaya et al. (4), Then and Felisa (5), Renwarin (6), Sitorus et al. (7), and Purwanto et al. (12). The results of this study have similarities with the research conducted by Hubner et al. (10), Haudi et al. (14), and Daud et al. (15).

Technology and information today have experienced very rapid development. Technology has opened as much access as possible for consumers to access a variety of information. The development of increasingly sophisticated technology accompanied by the use of the internet in the marketing process makes it easier for users to interact with each other. This has a positive impact on business people where the delivery of information can be done quickly, with a wide reach, and does not require expensive costs. Consumers are facilitated in finding information about the desired product without having to meet face to face. Word-of-mouth (WOM) is a phenomenon in the field of marketing, because nowadays consumers are always looking for references and trusting opinions in the community about a product. It is undeniable that the power of word-of-mouth has a big influence in developing the image of the goals to be conveyed by the company. Many studies have found how WOM exerts a stronger influence than other traditional communication media such as advertising or editorial recommendations. WOM is considered superior because information is more reliable. eWOM is a communication medium for sharing information about a product or service that has been consumed between consumers who initially did not know each other and met previously that were delivered electronically. eWOM has been shown to play a major role in consumer purchasing decisions by influencing consumer choices. Existing studies outline the statement that eWOM has become a permanent element of the online marketing mix by making a major contribution to consumers' online purchasing decisions. By using eWOM companies can benefit at low costs. With the internet and online media, consumers are now able to influence other consumers with their opinions and experiences. The possibility that people have received the information also helps spread the message on social media.

## Conclusion and Opinion

The various trends and benefits that exist in the health 4.0 era should make the health industry more initiative and competitive to participate in developing a similar system in their respective service facilities. However, this certainly cannot be separated from various obstacles and challenges in the implementation process. In the next part of this white paper.E-WOM has a positive and significant effect on Visiting Interest. An increase in the E-WOM variable will encourage a significant increase in the visiting interest variable and a decrease in the E-WOM variable will encourage a significant increase in the visiting interest variable. Viral marketing has a positive and significant effect on visiting interest. An increase in the Viral marketing variable will encourage a significant increase in the Visiting Interest variable and a decrease in the Viral marketing variable will encourage a significant increase in the Visiting Interest variable. E-WOM has a positive and significant effect on visiting decisions. An increase in the E-WOM variable will encourage a significant increase in the visiting decision variable and a decrease in the E-WOM variable will encourage a significant increase in the visiting decision variable. Viral marketing has a positive and significant impact on visiting decisions. An increase in the Viral marketing variable will encourage a significant increase in the visiting decision variable and a decrease in the Viral marketing variable will encourage a significant increase in the visiting decision variable. Interest in visiting has a positive and significant effect on visiting decisions. An increase in the visiting interest variable will encourage a significant increase in the visiting decision variable and a decrease in the visiting interest variable will encourage a significant increase in the visiting decision variable.

## Author Contributions

All authors listed have made a substantial, direct, and intellectual contribution to the work and approved it for publication.

## Conflict of Interest

The authors declare that the research was conducted in the absence of any commercial or financial relationships that could be construed as a potential conflict of interest.

## Publisher's Note

All claims expressed in this article are solely those of the authors and do not necessarily represent those of their affiliated organizations, or those of the publisher, the editors and the reviewers. Any product that may be evaluated in this article, or claim that may be made by its manufacturer, is not guaranteed or endorsed by the publisher.
